# Supplementary material for: Activity-dependent synapse elimination requires caspase-3 activation
Source: eLife. 2025 Jun 10;13:RP101779. doi: 10.7554/eLife.101779 (PMC12151539; doi:10.7554/eLife.101779)
Supplement: Source code 1. [file elife-101779-code1.zip › Source Code 1.docx]

**Computer code (Matlab Mathworks)**

***% The following is an image processing function that takes signal from both channels of a dLGN***

***% image and signal and background masks to calculate percentage overlap. The thresholds***

***% increase by 2.5% per step. If start_threshold_num = 4 and threshold_num = 7, then the***

***% starting threshold is 4*2.5 = 10%, and increase by 2.5% each time for 6 more thresholds,***

***% and the highest threshold is 25%.***

function [ipsi_area_vector, overlap_area_vector, roi_area, section_overlap_fraction_vector, log_ratio_vector, log_ratio_variance] = dLGNprocessing(contra_raw_int, ipsi_raw_int, sg_mask, bg_mask)

threshold_num = 7;

start_threshold_num = 4;

contra_raw = double(contra_raw_int);

ipsi_raw = double(ipsi_raw_int);

sg_indicator = sg_mask > 0;

bg_indicator = bg_mask > 0;

roi_area = sum(sum(sg_indicator));

% create indicator matrices using masks for background region and signal

% region.

contra_bg = mean(contra_raw(bg_indicator));

ipsi_bg = mean(ipsi_raw(bg_indicator));

% calculate background values of contra and ipsi channels.

contra_bg_rmv = contra_raw - contra_bg;

contra_bg_rmv_crt = contra_bg_rmv .* (contra_bg_rmv > 0);

contra_bg_rmv_crt_norm = contra_bg_rmv_crt / max(contra_bg_rmv_crt(sg_indicator));

ipsi_bg_rmv = ipsi_raw - ipsi_bg;

ipsi_bg_rmv_crt = ipsi_bg_rmv .* (ipsi_bg_rmv > 0);

ipsi_bg_rmv_crt_norm = ipsi_bg_rmv_crt / max(ipsi_bg_rmv_crt(sg_indicator));

% create versions of both channels that have background subtracted, with

% values lower than background set to zero, and also normalized to the max

% values in the signal regions.

overlap_area_vector = zeros(1,threshold_num);

ipsi_area_vector = zeros(1,threshold_num);

% create a vector recording overlap areas. The element in the nth column

% is the area in the region of interest that has values from both channels

% exceeding n*2.5 percent of the max value.

for n = start_threshold_num:(start_threshold_num + threshold_num - 1)

overlap_area_vector(n - start_threshold_num + 1) = sum(sum( (contra_bg_rmv_crt_norm >= n*0.025) & (ipsi_bg_rmv_crt_norm >= n*0.025) & sg_indicator ));

ipsi_area_vector(n - start_threshold_num + 1) = sum(sum((ipsi_bg_rmv_crt_norm >= n*0.025) & sg_indicator));

end

section_overlap_fraction_vector = overlap_area_vector / roi_area;

contra_non_zero = contra_raw + (contra_raw == 0);

ipsi_non_zero = ipsi_raw + (ipsi_raw == 0);

contra_norm = contra_non_zero / max(contra_non_zero(sg_indicator));

ipsi_norm = ipsi_non_zero / max(ipsi_non_zero(sg_indicator));

contra_to_ipsi_log_ratio = log( contra_norm ./ ipsi_norm );

log_ratio_vector = contra_to_ipsi_log_ratio(sg_indicator);

log_ratio_variance = var(log_ratio_vector);

end

***% The following is a script that calls the function above and calculate percentage overlap for a group of***

***% animals. This script assumes that the image files are named by first labeling whether is a signal mask,***

***% a background mask, or a raw image of contra/ipsilateral signal, then specifying genotype, age, side of***

***% the brain, then specifying animal number and section number. The user can specify from which***

***% section the analysis starts and how many sections to analyze. The percentage overlap across multiple***

***% thresholds in multiple animals will be stored in total_overlap_fraction_vector.***

sgmask_file_format = 'sgmask_%s_%s_%s_%d_%d.tif';

bgmask_file_format = 'bgmask_%s_%s_%s_%d_%d.tif';

contra_file_format = 'contra_%s_%s_%s_%d_%d.tif';

ipsi_file_format = 'ipsi_%s_%s_%s_%d_%d.tif';

threshold_num = 7;

genotype = 'homko';

age = 'p10';

side = 'l';

animal_number = 6;

section_number = 7;

start_section = 2;

section_overlap_fraction_matrix = zeros(section_number, threshold_num);

section_overlap_area_matrix = zeros(section_number, threshold_num);

section_ipsi_area_matrix = zeros(section_number, threshold_num);

section_roi_area_vector = zeros(section_number, 1);

pooled_log_ratio_vector = [];

log_ratio_variance_vector = zeros(section_number, 1);

for n = start_section:(start_section + section_number - 1)

sgmask_file = sprintf(sgmask_file_format, genotype, age, side, animal_number, n);

bgmask_file = sprintf(bgmask_file_format, genotype, age, side, animal_number, n);

contra_file = sprintf(contra_file_format, genotype, age, side, animal_number, n);

ipsi_file = sprintf(ipsi_file_format, genotype, age, side, animal_number, n);

[section_ipsi_area_matrix(n,:), section_overlap_area_matrix(n,:), section_roi_area_vector(n), section_overlap_fraction_matrix(n,:), log_ratio_vector, log_ratio_variance_vector(n)]...

= dLGNprocessing(imread(contra_file), imread(ipsi_file), imread(sgmask_file), imread(bgmask_file));

pooled_log_ratio_vector = [pooled_log_ratio_vector; log_ratio_vector];

end

total_overlap_volume_vector = sum(section_overlap_area_matrix);

total_roi_volume = sum(section_roi_area_vector);

total_ipsi_volume_vector = sum(section_ipsi_area_matrix);

total_overlap_fraction_vector = total_overlap_volume_vector / total_roi_volume;

total_overlap_of_ipsi_fraction_vector = total_overlap_volume_vector ./ total_ipsi_volume_vector;
